# Nanoemulsion-based colistin for pulmonary delivery: Enhanced antibacterial efficacy against *Acinetobacter baumannii*

**DOI:** 10.1007/s13346-026-02083-z

**Published:** 2026-03-30

**Authors:** Marta Martínez-Guitián, Lucía Sanjurjo, Juan Carlos Vázquez-Ucha, Andrea Muras, Alejandro Beceiro, José Crecente-Campo, María José Alonso

**Affiliations:** 1 Department of Health of Sciences, University of A Coruña, 15006 A Coruña, Spain; 2 Center for Research in Molecular Medicine and Chronic Diseases (CIMUS), University of Santiago de Compostela, 15782 Santiago de Compostela, Spain; 3 Health Research Institute of Santiago de Compostela (IDIS), 15706 Santiago de Compostela, Spain; 4 Microbiology Service and Institute of Biomedical Research of A Coruña (INIBIC), A Coruña University Hospital Complex (CHUAC), 15006 A Coruña, Spain; 5 Department of Pharmacology Pharmacy and Pharmaceutical Technology, School of Pharmacy, University of Santiago de Compostela, 15782 Santiago de Compostela, Spain

**Keywords:** *Acinetobacter baumannii*, Colistin, Nanoemulsion, In vivo model, Pneumonia

## Abstract

Infections caused by *Acinetobacter baumannii*, which frequently result in pneumonia and/or bacteraemia, represent a significant clinical challenge. Colistin, an antimicrobial peptide used as a last-resort therapy due to its high toxicity, is employed to treat severe infections, i.e. those caused by *A. baumannii*. The aim of this study was to develop a colistin-loaded nanoformulation (COL-NE) capable of targeting infected cells in the lung, thereby enhancing antibacterial efficacy while reducing toxicity. After screening multiple formulations, an optimized colistin nanoemulsion (COL-NE) was developed, exhibiting a particle size of 180 nm and a 1–4-fold reduction in MIC against *A. baumannii* compared to free colistin. The nanoemulsion also displayed significant antibiofilm activity, enhanced cellular penetration, and a 27–45% reduction in in vitro toxicity relative to colistin. Notably, following intratracheal administration, COL-NE improved the elimination of intracellular bacteria in macrophages through passive targeting while maintaining activity against extracellular bacteria. In a murine pneumonia model, COL-NE reduced lung bacterial burden by 2 log₁₀ CFU/mL compared with untreated controls and by 1.25 log₁₀ CFU/mL relative to colistin-treated mice. These findings highlight the potential of colistin-loaded nanoemulsions as a promising therapeutic strategy against *A. baumannii* infections, enhancing antibacterial efficacy while mitigating colistin-associated toxicity.

**Graphical abstract:**

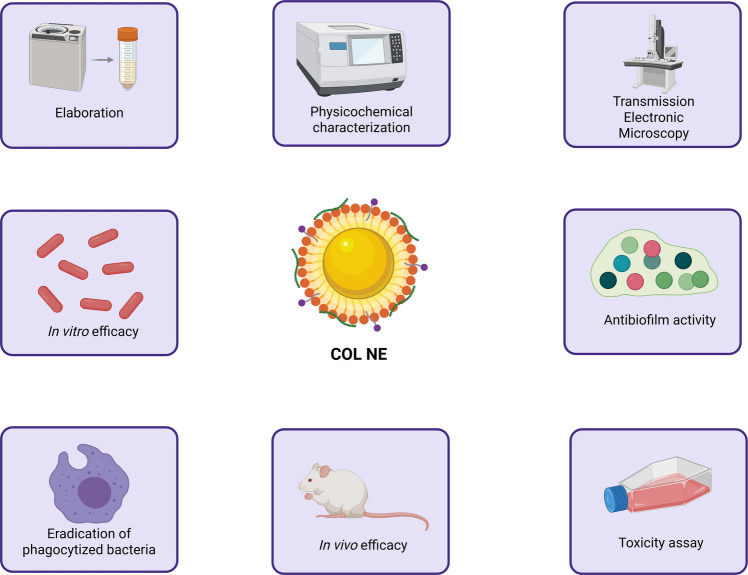

**Supplementary Information:**

The online version contains supplementary material available at 10.1007/s13346-026-02083-z.

## Introduction

*Acinetobacter baumannii* is a significant hospital-acquired pathogen known for its ability to survive in healthcare settings and resist various antibiotics. It commonly causes serious infections, such as pneumonia, skin and soft tissue infections, and bloodstream infections, especially in vulnerable patients. Carbapenems are the primary antibiotics used to treat these infections [1]. However, infections caused by carbapenem-resistant *A. baumannii* (CRAB) isolates are alarmingly increasing in last decades; around of one-third of *A. baumannii* isolates collected in European Union display carbapenem-resistance and have low susceptibility rates to other antimicrobial option [2, 3]. The prognosis of patients infected by CRAB is frequently very poor and mortality rates are higher than for infections caused by carbapenem-susceptible isolates [4]. For all these reasons, the World Health Organization has classified CRAB under the Critical Category, requiring urgent new antibiotic treatments [5].

Colistin (polymyxin E) is a last-resort antibiotic used against Gram-negative bacteria like *A. baumannii*. It kills bacteria by targeting their negatively charged outer membrane, displacing calcium and magnesium ions, which disrupts membrane stability and leads to cell lysis and death [6, 7]. This polymyxin can be administered as an inactive prodrug, sodium colistimethate (CMS), when it is used intravenously [8]. CMS is hydrolysed in vivo to regain its activity and presents fewer side effects than colistin, however, it also displays lower antimicrobial activity [9].

Colistin, approved by the FDA in 1959, fell out of use by the 1970 s due to its high toxicity. However, rising antibiotic resistance prompted its reintroduction into clinical practice in the mid-1990s [6, 10]. Colistin’s main adverse effects are nephrotoxicity and neurotoxicity. In the kidney, it enters proximal tubule cells via megalin-mediated endocytosis and PEPT2/OCTN2 transporters owing to its polycationic nature. Once internalised, it causes mitochondrial dysfunction and endoplasmic reticulum (ER) stress, which activate apoptotic pathways (death receptor, mitochondrial, ER). This leads to dose- and time-dependent nephrotoxicity, particularly in the proximal tubule, with an incidence of 20–50%, influenced by patient risk factors and dosing [11, 12]. Inhaled dry powder colistin reduces systemic toxicity and improves lung infection treatment. However, current nebulised therapy is empirical and may increase the risk of pulmonary toxicity [13–15]. In the lungs, colistin is internalized through PEPT1 and PEPT2 transporters, where it induces oxidative stress, mitochondrial dysfunction, and apoptosis, contributing to tissue toxicity. Additionally, as a cationic polypeptide, colistin can electrostatically interact with pulmonary surfactants in the epithelial lining fluid and with mucin in the sputum [14, 16]. Nanotechnology offers valuable opportunities to improve complex drugs with unfavourable physicochemical and biological properties, enhancing their efficacy while reducing toxicity [17]. The choice of a nanoemulsion as a delivery vehicle for colistin was motivated by its potential to partially mask the positive charge by retaining the molecule to the nanoemulsion surface. Neutralization of colistin’s positive charge is expected to limit its uptake by eukaryotic cells and thereby mitigate dose-dependent toxicity. Despite these theoretical advantages, as well as the simplicity of production and favourable storage stability, this delivery approach has been scarcely explored for antimicrobial peptides [18, 19]. To date, only one study has investigated a colistin-loaded nanoemulsion (like colistin methanesulfonate, CMS) against *A. baumannii* in vivo. Although that study lacked an appropriate control, the authors suggested that the formulation could enable longer dosing intervals and lower doses while maintaining therapeutic efficacy [20, 21].

Hence, building on our laboratory’s experience in the encapsulation of peptides within nanoemulsions and nanocapsules [22–24], we proposed a colistin-loaded nanoemulsion (COL-NE) as a potential therapeutic approach for bacterial lung infections. Our objective was to design a delivery system that maintained maximum simplicity while enabling convenient inhalation-based administration. The developed COL-NE was thoroughly characterized, and its in vitro and in vivo performance was evaluated in terms of efficacy and toxicity following pulmonary administration. To our knowledge, this is the first study to assess the in vivo efficacy of a colistin sulphate nanoemulsion against the multidrug-resistant pathogen *A. baumannii*.

## Material and methods

### Reagents, bacterial strains, and cell cultures

Colistin sulphate was acquired to Fagron (Barcelona, Spain); LipoidP45 (lecithin fraction with 45% phosphatidylcholine) was obtained from Lipoid GmbH (Ludwigshafen, Germany); D-α-Tocopherol polyethylene glycol 1000 succinate (TPGS) was obtained from Antares Health Products (Jonesborough, USA); and caprylic/capric triglyceride (Miglyol^®^812-N) was obtained from BG Oleochemicals (Barcelona, Spain).

Reference and clinical strains of *A. baumannii* included in this study are described in Table 1 and were cultured at 37ºC in Luria–Bertani medium (LB) and were maintained in LB with 10% glycerol, at −80ºC, until analysis. The *A. baumannii* ATCC19606 strain was employed for the assays, as it constitutes the type strain, that is, the representative strain of the specie.

**Table 1:** Description of the different components of the nanoemulsion

Compound	Function	Purpose	% w/v	% relative to solutes^1^
Lipoid P45	Emulsifier and stabilizer	Promotes nanosized droplet formation and biocompatibility	2%	18.8%
TPGS (D-α-Tocopheryl polyethylene licol 1000 succinate)	Surfactant and solubility enhancer	Stabilizes nanoemulsion and extends circulation time	0.625%	5.9%
Miglyol^®^812-N	Oil phase	Biodegradable and well tolerated	6.02%	56.6%
Colistin sulphate	Active antibiotic	Antibiotic of last resort therapeutic	2%	18.8%

^**1**^ The percentage relative to the solutes shows what fraction of the total solutes each component represents, without counting the solvents (water and ethanol)

A549 human alveolar epithelial cells, CMT167 murine alveolar epithelial cells and RAW264.7 murine macrophages were purchased from American Type Culture Collection (Manassas, USA). Cell lines were maintained at 37ºC in the presence of 5% CO_2_ in Dulbecco’s modified Eagle medium (DMEM, Sigma-Aldrich, San Luis, USA) supplemented with 10% of heat-inactivated foetal bovine serum (FBS, Thermo-Fisher, Waltham, USA) and 1% penicillin (10,000 U/mL)/streptomycin (10,000 µg/mL) (Thermo-Fisher, Waltham, USA).

### Preparation of nanoemulsions

Colistin-loaded nanoemulsion (COL-NE) and drug-free nanoemulsion (blank-NE) were prepared by solvent displacement technique following the procedure previously described by Abellán-Pose et al. [25]. Briefly, the organic phase was prepared by mixing 10 mg of Lipoid P45 in 0.5 mL ethanol, 3.125 mg of TPGS in 62.5 µL ethanol, and 31.7 µL of Miglyol^®^ 812 N. This mixture was immediately added, using a micropipette, to 4.4 mL of ultrapure water at room temperature under magnetic stirring, resulting in the instantaneous formation of the nanoemulsion. Previously, 250 µL of colistin solution (40 mg/mL in water) was incorporated into the aqueous phase of the pre-formed nanoemulsion. Finally, COL-NE was isolated and concentrated by ultracentrifugation at 29,500 × *g* for 1 h at 15 ºC in an OptimaTM L–90 K ultracentrifuge (Beckmann Coulter, Fullerton, USA) to final concentration of 1 mg/mL.

### Encapsulation efficiency of colistin and drug release profile

Encapsulation efficiency (E.E.) of colistin was determined using both a direct and an indirect method, as follow:

$${E.E}_{direct}\,\%=\frac{drug \,in \,isolated \,nanoemulsion}{drug\, in \,non \,isolated \,nanoemulsion}\times 100$$

$${E.E}_{indirect}\,\%=\frac{drug \,in \,non \,isolated \,nanoemulsion-drug\, in \,infranant}{drug \,in \,non \,isolated \,nanoemulsion}\times 100$$

For this evaluation, aliquots of all these fractions were dissolved 1:4 with acetonitrile and shaken at 1700 rpm for 40 min at room temperature (RT) in a shaker Eppendorf MixMate 1.5/2.0 mL (Eppendorf, Hamburg, Germany). Then, samples were diluted in a mixture of acetonitrile and ultrapure water (1:3:1, v/v) and were analysed by ultra-performance liquid chromatography (UPLC). The UPLC system consisted of a Waters Acquity H-Class UPLC (Waters Corporation, Milford, USA) equipped with a UV detector set at 200 nm and a reverse phase Phenomenex Aeris™ XB-C18 column (3.6 µm, 100 × 2.1 mm, 200 Å; Phenomenex, California, USA). For this purpose, 4 μL of each sample at 20ºC were injected and the flow rate was set to 0.2 mL/min. The gradient was obtained by mixing two mobile phases: 0.1% formic acid in ultrapure water (phase A) and 0.1% formic acid in acetonitrile (phase B). Standard calibration curves of colistin were linear in the range of 0.0125–0.4 mg/mL (r^2^ = 0.9994).

For the drug release study, the formulation was dispersed in PBS (pH 7.4) to simulate physiological conditions and incubated at 37°C under agitation. Aliquots were collected at 0, 3, 8, and 24 h and analysed by UPLC-UV/Vis using the previously described method. To quantify only the free drug fraction, samples were ultracentrifuged under the conditions specified.

### Physicochemical characterization

The size and the polydispersity index (PDI) of nanoemulsions were analysed by photon correlation spectroscopy (PCS) and the ζ-potential was determined by laser Doppler anemometry (LDA), after appropriate dilution in ultrapure water. The PCS and LDA analysis were performed, in triplicate, using a Zetasizer Nano Pro (Malvern Instruments, Malvern, UK) (Table 1).

The morphology of nanoemulsions were evaluated by transmission electron microscopy (TEM) using a JEOL JEM-2010 microscope (Jeol, Tokyo, Japan).

### In vitro stability assays

To assess storage stability, the size, polydispersity index (PDI), ζ-potential, and encapsulation efficiency of the nanoemulsions were evaluated at predetermined time points, with all samples stored at 4ºC. Additionally, stability assays were conducted in diverse media, intended for in vitro assays: LB, Mueller Hinton-II adjusted cations broth (MH-II, Millipore, Sigma-Aldrich, San Luis, USA) and Hank’s balanced salt solution (HBSS, Gibco) at 37ºC for 24 h. Physicochemical properties of nanoemulsions were assessed by PCS and LDA analysis using a Zetasizer Nano Pro and the colistin concentration was tested by UPLC-UV/Vis.

### Antimicrobial activity against *A. baumannii*

The antimicrobial activity of COL-NE compared with those of free colistin was tested against both reference and carbapenem-resistant clinical *A. baumannii* strains. Colistin, COL-NE and blank-NE minimum inhibitory concentrations (MIC) were determined by broth microdilution in MH-II medium, following CLSI recommendations [26].

Time-kill assays at fixed concentration (0.5 mg/L) of blank-NE, COL-NE and free colistin were performed to measure the in vitro bactericidal activity, as indicated previously [27]. Colony forming units (CFU) were determined at 2, 4, 8 and 24 h by plating onto LB agar plates and incubating at 37ºC for 24 h.

### Transmission Electronic Microscopy (TEM)

Bacterial cells were cultured to an OD₆₀₀ of 0.7, then treated with colistin and nanoemulsions (0.5 mg/L) and incubated at 37ºC for 4 h. After centrifugation at 3000 × *g* for 15 min at 4ºC and washing with sodium cacodylate buffer (0.1 M), cells were fixed in 2.5% glutaraldehyde. Then, 10 µL of the sample were applied to copper TEM grids, negatively stained with 2% phosphotungstic acid, and imaged at 200 kV using a JEOL JEM-2010 transmission electron microscope.

### Biofilm production assay

A biofilm production test was conducted using a modified Amsterdam Active Attachment model with glass coverslips to compare biofilm formation under different treatments, as described previously [28]. After 6 h of bacterial growth in LB medium, colistin and nanoemulsions (1 mg/L) were added, with untreated wells serving as controls. Following 24 h of incubation at 37ºC, coverslips were stained with 0.04% crystal violet (Biomérieux, Marcy-l'Étoile, France), incubated for 20 min at RT, and the bound dye was released using 33% acetic acid. Biofilm formation was quantified by measuring absorbance at OD₆₀₀ [29].

### Effect of nanoemulsion in eradication of phagocytized bacteria

The penetration capacity of nanoemulsions in macrophages and the eradication capacity of opsonised bacteria were evaluated in RAW 264.7 macrophages. 2 × 10^4^ cells/well were infected with 10^7^ bacteria per well and incubated for 3 h in HBSS glucose free medium. After that, macrophages were treated with colistin, COL-NE, or blank-NE (2 mg/L) for 4 h. Then, cells were treated for 2 h with gentamicin (256 mg/L), washed and lysed with 0.5% sodium deoxycholate to evaluate bacterial eradication. Lysate dilutions were plated on LB agar and incubated at 37ºC for 24 h. CFU were then counted to assess the number of bacteria that survived intracellular treatment [30].

### Toxicity assay in alveolar epithelial cells

A cytotoxicity assay was performed in A549 human alveolar epithelial cells to evaluate the toxicity of COL-NE versus colistin. Cells were seeded onto 96 well-plates (8 × 10^3^ cells/well) in HBSS medium and treated with 100 mg/L of each formulation for 24 h. The viability of treated cells was examined with the Cell Counting Kit-8 (CCK-8, Sigma–Aldrich), following the manufacturer’s instructions.

On the other hand, an apoptosis assay was conducted in CMT167 murine alveolar epithelial cells. Cells were seeded onto 24 well-plate (10^5^ cells/well) in HBSS medium and treated with 100 mg/L of each formulation for 24 h. Supernatants and detached cells were collected, centrifuged, and stained with propidium iodide (1:1000 in PBS). Samples were then analysed by flow cytometry using a FACS Calibur Flow Cytometer (BD Bioscience, San Jose, USA), with at least three independent replicates of 5,000 events each.

### Efficacy of treatments in murine pneumonia model

A murine pneumonia model was used to examine the efficacy in vivo of nanoemulsions against an infection caused by *A. baumannii* ATCC19606 strain. BALB/c male mice from 12 weeks old were housed in regulation cages with food and water ad libitum. All mice were maintained in the specific-pathogen-free facility at the Centro Tecnolóxico de Formación da Xerencia de Xestión Integrada A Coruña (CTF-XXIAC). The study was approved by the Ethics and Clinical Research Committee (CHUAC, project code P169/15002/2023/005; Spain).

Groups of 7 mice were immunosuppressed as described previously [31, 32]. Then, mice were intratracheally inoculated with bacterial suspension containing 4 × 10^7^ CFU, as described Vázquez-Uchaet al.[33]. Two doses (2 mg/kg) of colistin and nanoemulsions were administered intratracheally with a maximum volume of 40 µL at 6 and 18 h after infection. Mice were sacrificed at 24 h after infection and lung samples were obtained and processed as previously indicated [34]. Dilutions of the lung samples were plated onto LB agar and incubated at 37ºC for 24 h. CFU were counted to determine the antimicrobial activity.

### Statistical significance

A minimum of three independent assays were performed, each with three technical replicates. ANOVA corrected with Tukey’s multiple comparisons tests were performed to evaluate the statistical significance of observed differences and *p* values of < 0.05 were considered statistically significant.

## Results and discussion

Despite its toxicity, the anti-infectious peptide colistin is being increasingly used due to the global spread of CRAB. The limited availability of the peptide in infected lung tissue under standard parenteral administration necessitates dose escalation, thereby amplifying toxicity. In this context, the use of nanocarriers for pulmonary administration is a promising alternative for facilitating the access of the drug to the targeted tissue, thereby expecting to improve [35, 36] both efficacy and safety.

### Physicochemical characterization and encapsulation efficiency of nanoemulsions

A nanoemulsion was selected based on our previous experience with the encapsulation of peptides in a nanoemulsion system. As outlined in the introduction, our aim was to develop a delivery carrier that would be as simple as possible while allowing for convenient administration by inhalation. The translational potential of such a formulation is considerable, owing to its straightforward fabrication process, biocompatibility, minimal need for emulsifiers and surfactants during preparation, and excellent storage stability. Although the formulation design was intentionally simple, it still required substantial optimization, which was made feasible by our prior expertise in peptide formulation.

Hence, a Miglyol^®^-based nanoemulsion was engineered using the solvent displacement technique, with Lipoid P45 serving as a stabilizer in combination with TPGS, a PEGylated derivative of vitamin E, which further enhances the stability of the nanoemulsion and helps to mask the positive charge of colistin [37, 38]. The proportion of each component was optimized through formulation screening guided by a target product profile that aimed to achieve adequate encapsulation efficiency, a low positive surface charge, and a small particle size.

**Fig. 1:**
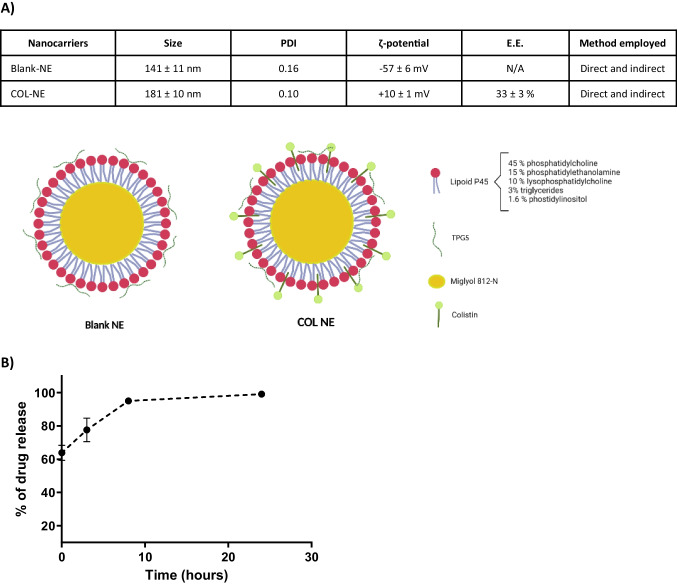
**A**) Physicochemical characteristics and efficacy of encapsulation of nanoemulsion used in this work. **B**) Drug release profile

**Fig. 2:**
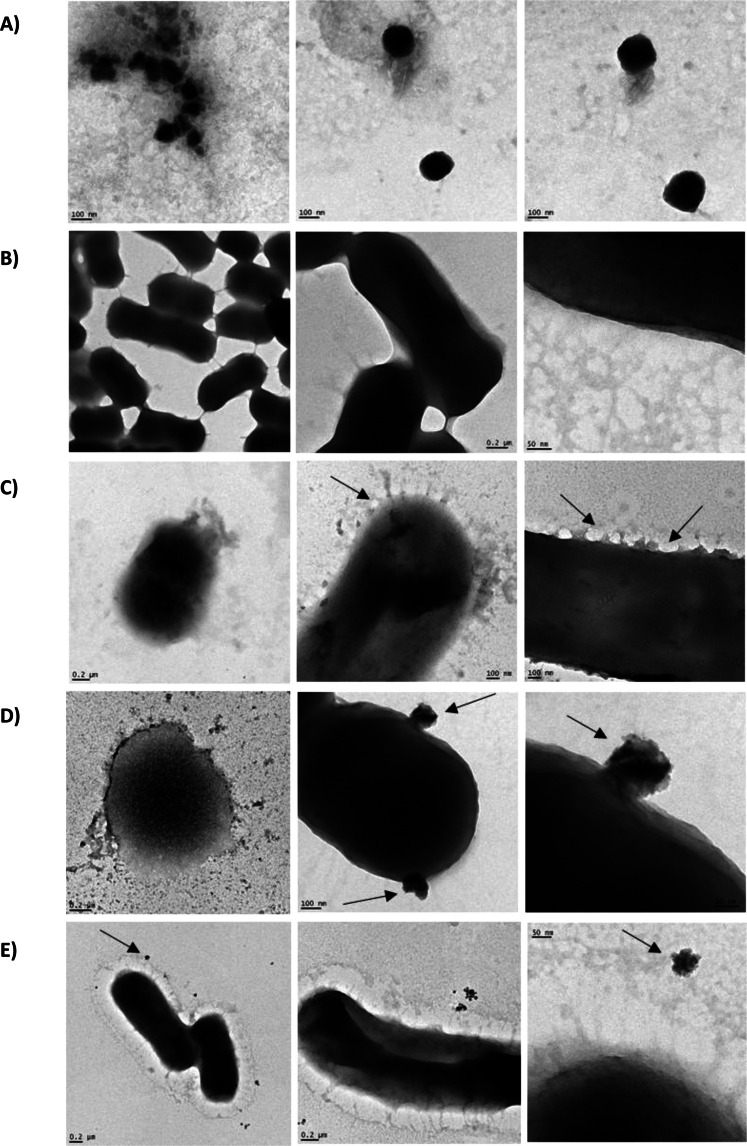
TEM images of nanoemulsions and their interaction with *A. baumannii* ATCC19606 strain. **A**) Colistin nanoemulsion; **B**) untreated *A. baumannii* strain; **C**) *A. baumannii* strain after colistin treatment. Broken membrane is indicated by arrows; **D**) *A. baumannii* strain after colistin nanoemulsion. Nanoemulsion is fused with the bacterial membrane; **E**) *A. baumannii* strain after blank nanoemulsion treatment

As shown in Table 1, Fig. 1A and Fig. 2A, the resulting COL-NE (1 mg/mL of colistin) exhibited a mean particle size of 181 ± 10 nm, a PDI of 0.10, a low positive zeta potential of + 10 ± 1 mV, and an encapsulation efficiency of 33%. These characteristics differed markedly from those of the blank nanoemulsion, which displayed a smaller particle size 141 ± 11 nm and a highly negative zeta potential (–57 ± 6 mV). Notably, the increase in particle size and the inversion of the zeta potential from negative to slightly positive were attributed to the incorporation of colistin and its intrinsic cationic nature.

The formulation was found to be stable for 24 h in various media (LB, MH-II, and glucose-free HBSS) and (Fig. S1, see online supplementary material). It exhibited a pronounced burst release (Fig. 1B), which is a typical profile for emulsions, where the release mechanism is governed by the diffusion of the drug from the oily phase into the surrounding aqueous medium. Furthermore, the presence of a fraction of free colistin in the formulation may be beneficial for rapid bacterial clearance during the initial hours, while the subsequent increased release will contribute to the bacterial clearance in the exponential phase of bacterial growth (2–12 h) [40].

The positive charge of colistin is essential for its antimicrobial action, as it enables electrostatic binding to the negatively charged lipid A of lipopolysaccharides (LPS), displacing divalent cations that stabilize adjacent LPS molecules. The *A. baumannii* outer membrane exhibits a net negative ζ-potential (−60.5 to −26.2 mV, depending on the strain), which becomes neutralized during colistin treatment [41]. Thus, the partial charge neutralization, maintaining a certain positive charge is therefore crucial for antibacterial efficacy but also contributes to toxicity through interactions with PEPT transporters in eukaryotic cells, leading to mitochondrial and endoplasmic reticulum dysfunction and apoptosis [11]. The moderate positive charge of COL-NE (+ 10 mV) is expected to preserve antibacterial activity while reducing transporter affinity and associated toxicity. Moreover, the initial burst release likely supports early bacterial clearance during the exponential growth phase without premature drug exhaustion.

Storage stability studies showed that the nanoemulsion remained stable for at least 8 months at 4ºC, maintaining consistent particle size, PDI, and ζ-potential. Colistin concentration (1 mg/mL) was preserved for at least 1 month under the same conditions (Fig. S2).

### In vitro assessment of antimicrobial activity

Antimicrobial activity assays were conducted to assess the bactericidal efficacy of nanoemulsions. The MICs of colistin, COL-NE, and blank-NE were determined against both clinical and reference strains of *A. baumannii* harbouring various antimicrobial resistance mechanisms. The MICs of colistin ranged from 0.5 to 2 mg/L. The blank-NE showed no antimicrobial activity (MIC > 256 mg/L) (Table 2). Like findings in other published studies, the MICs of COL-NE closely match those of free colistin. For instance, the CMS-loaded nanoemulsion developed by Vairo et al. showed a 1- to twofold MIC reduction compared to free CMS [42]. Similarly, colistin-loaded human albumin nanoparticles showed a modest 1-fold MIC reduction compared to colistin alone [43, 44]. For the *A. baumannii* ATCC17978 strain, we observed a 4-fold decrease in the MIC compared to that of free colistin. This reduction is likely attributable to genetic differences between the strains.

**Table 2:** Description of *A. baumannii* strains and MICs of colistin and nanoemulsions

*A. baumannii* strains	MIC (mg/L) in MH-II adjusted cations medium			Description
	Colistin sulphate	COL-NE	Blank-NE	
ATCC19606	2	1	> 256	*A. baumannii* type strain
ATCC17978	1	0.25	> 256	*A. baumannii* reference strain
Clinical strain ABRIM	2	0.5	> 256	*A. baumannii* clinical isolate
Clinical strain AbH12O-A2	2	1	> 256	Cephalosporin resistant and CRAB clinical isolate producing OXA-65
Clinical strain 001	1	0.5	> 256	CRAB clinical isolate producing OXA-143
Clinical strain 002	2	1	> 256	CRAB clinical isolate producing OXA-24/40
Cinical strain 003	2	2	> 256	CRAB clinical isolate producing OXA-23
Clinical strain 004	2	2	> 256	CRAB clinical isolate producing OXA-58

**Fig. 3:**
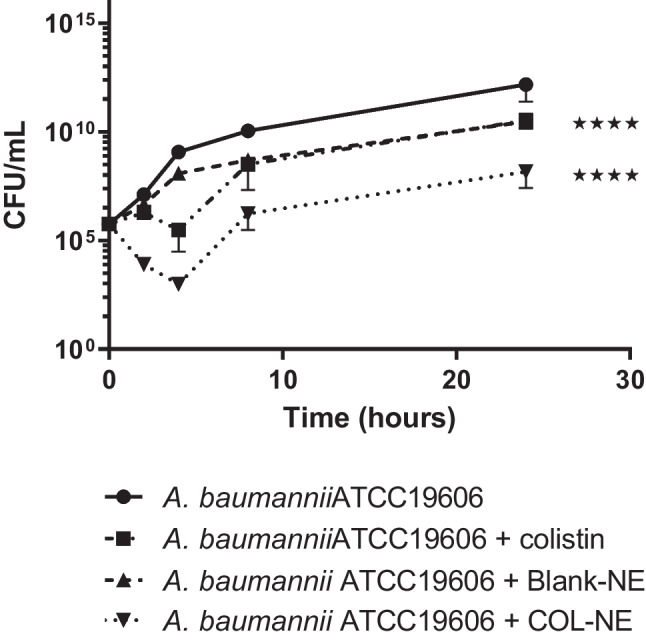
Antimicrobial activity of colistin and nanoemulsions in LB medium. Antibiotic molecules were added at 0.5 mg/L. ANOVA corrected with Tukey’s multiple comparisons test was used to validate the experimental data (***, *p* < 0.001)

To further assess the impact of COL-NE on bacterial growth, time-kill assays were conducted in LB medium (Fig. 3). The results showed that COL-NE significantly outperformed free colistin against *A. baumannii* ATCC19606. At 0.5 mg/L, COL-NE reduced bacterial counts by 6 and 4 log₁₀ CFU/mL at 4 and 8 h, compared to 3.6 and 1.5 log₁₀ for free colistin. This enhanced antimicrobial effect lasted at least 24 h (***p < 0.001). On the other hand, we observed a slight antimicrobial effect for Blank-NE, which became evident after 8 h and increased at 24 h. This observation could be attributed to a potential antimicrobial effect of the vehicle. Nevertheless, the MIC values obtained were > 256 mg/L, indicating that this effect cannot be considered true antimicrobial activity. The differences between assays may be due to methodological variations, since time–kill curves are performed under orbital agitation, while microdilution assays are carried out under static conditions. While these findings are consistent with a previous study using colistin and niclosamide nanoemulsions against *Salmonella*, which demonstrated a reduction in bacterial load of over 2 log_10_ CFU/mL compared to the free antibiotic [45], this study represents the first report of a time-kill assay for colistin formulated as a nanosystem against *A. baumannii*.

The explanation to these positive results was found on the TEM analysis of the interaction of COL-NE with bacteria. Compared to untreated controls (Fig. 2B), colistin was observed to damage the bacterial OM by forming pores (Fig. 2C), leading to cell lysis, which supports its enhanced antibacterial activity [6]. Fusion between nanoemulsions and the OM has been observed in the images obtained by TEM. However, we found that, unlike the COL-NE, the blank-NE keep the bacterial OM intact (Figs. 2D and E). Previous studies by Nicolosy et al*.* and Pushparaj-Selvadoss et al*.* demonstrated that lipid-based nanocarriers have fusogenic properties, allowing them to bypass the OM of Gram-negative bacteria and deliver antibiotics closer to the periplasmic space, enhancing their activity at the bacterial cytoplasmic level [46, 47]. On the other hand, colistin does not only bind to LPS in the OM but also to the forming LPS in the cytoplasmic membrane (CM). As in the OM, colistin binding to LPS results in the disruption of the CM, culminating in the loss of cytoplasmic contents and bacterial death [48]. These results have led to the conclusion that COL-NE facilitate the delivery of colistin to the cytoplasm thus acting more efficiently.

**Fig. 4:**
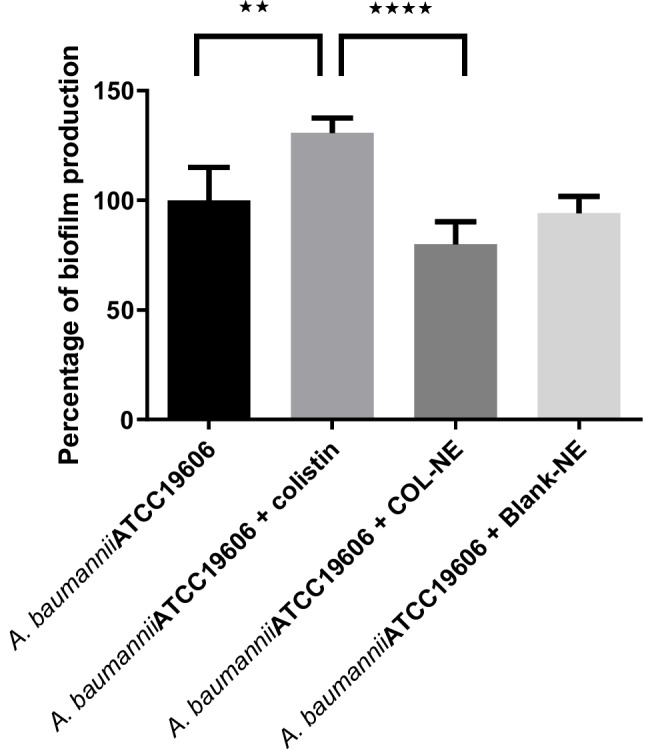
Quantification of biofilm formation by the *A. baumannii* ATCC19606 strain and *A. baumannii* ATCC19606 strain treated with colistin sulphate or nanoemulsions at 1 mg/L. ANOVA corrected with Tukey’s multiple comparisons test was used to validate the experimental data (***, *p* < 0.001)

A critical barrier for bacteria to resist antibiotics efficacy is the formation of a biofilm, consisting of a hydrated polymeric matrix, holding highly structured community of bacterial microcolonies. Biofilm is particularly important mechanism responsible for the high virulence of Gram-negative pathogens, including *A. baumannii* [49, 50]. In this work, we evaluated the biofilm formation ability of *A. baumannii* ATCC19606 strain in presence of colistin, COL-NE or blank-NE (Fig. 4). Antibiotic and nanoemulsions were added at 1 mg/L onto a pre-formed biofilm. A concentration of 1 mg/L was chosen to assess antibiofilm activity because it is below the MIC yet high enough to reveal colistin’s unique ability to modulate genes linked to biofilm overproduction—an effect not observable at very low concentrations and lost when the biofilm is fully eradicated at higher doses. The results showed that, in the presence of free colistin, *A. baumannii* ATCC19606 strain showed an increase of a 30.75% biofilm production (130.72 ± 6.8%) compared to the untreated bacteria (**p < 0.01). These findings align with those reported by Shenkutie et al. who found that colistin upregulated 28 of 44 differentially expressed genes in biofilm cells, enhancing biofilm formation and promoting mutations that contribute to antibiotic resistance [51, 52]. In contrast to these negative results, the treatment with COL-NE led to a reduction of 20.01% in biofilm production (79.96 ± 17.3%) as compared to those in absence of treatment. This effect was even more remarkable when compared to that of the free antibiotic, allowing for a reduction 50.76% (****p < 0.0001) in biofilm production. In contrast blank-NE did not present antibiofilm activity (94.06 ± 7.8%). A similar study conducted with colistin-loaded albumin nanoparticles showed a 30% reduction in biofilm formation compared to free colistin, thus showing the out-performing capacity of the COL-NE [44]. Other works exploring antibiofilm activity of colistin-loaded nanocarriers have been focused on *Pseudomonas aeruginosa* strains [53, 54]. The superior anti-biofilm effect achieved with COL-NE could be related to its composition and capacity of interaction of the NE across the biofilm metabolically active cells.

The contradictory effect observed for free colistin in terms of biofilm production has been associated to its inability to target the outermost layers of the biofilm, where the most metabolically active cells are located [55, 56]. In this regard, it has been described that the interaction between the phosphatidylinositol moieties from lipid-based carriers and bacterial biofilms, specifically with the bacterial glycocalyx, a polysaccharide exocellular slime which resists antibiotic penetration is the key in their mechanism of action [57, 58]. Therefore, in agreement with this previous reports, we could infer that COL-NE could achieve a better eradication of the biofilm due to their interaction with the different extracts that make up the biofilm [53].

### in vitro evaluation of infection eradication and toxicity in the lung

Alveolar macrophages (AMs) are essential components of the pulmonary immune system, serving as the first line of defence against respiratory pathogens, including *A. baumannii*. They efficiently phagocytose this bacterium shortly after exposure. However, their bactericidal capacity is limited compared to that of other immune cells, such as neutrophils. While AMs play a crucial role in initiating immune responses and recruiting other immune cells, they are less effective in eradicating *A. baumannii*, leading to challenges in clearing this pathogen from the lungs [59, 60]. AM produce reactive oxygen species in response to intracellular *A. baumannii* but could not eliminate it completely, because this pathogen present the catalase activity upregulated and is more resistant to the toxicity caused by H_2_O_2_ [60, 61]. It should be noted that colistin has been shown to penetrate poorly into host cells, whereas nanocarriers are easily captured by macrophages [61, 62].

**Fig. 5:**
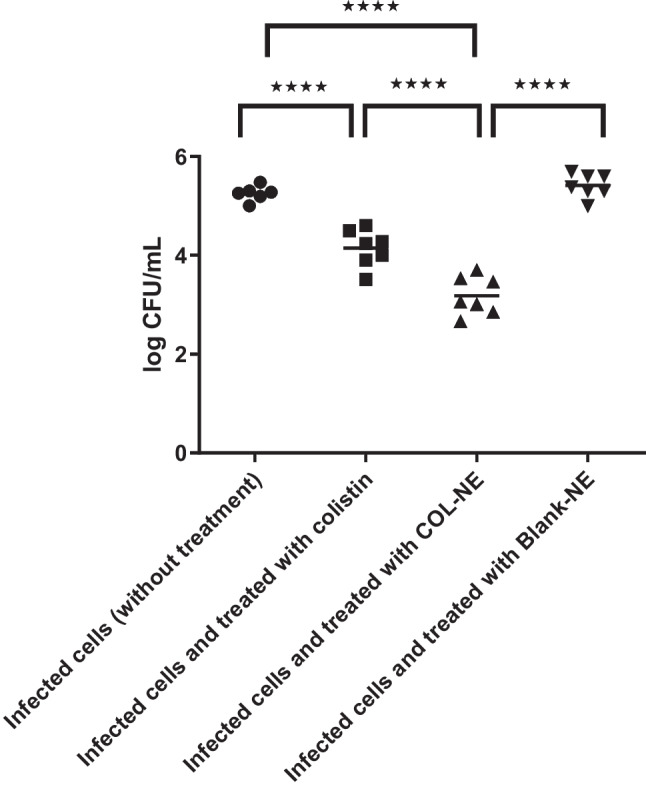
Effect of nanoemulsion in eradication of phagocytized bacteria. Infection of RAW264.7 murine macrophages with *A. baumannii* ATCC19606 strain and treatment with colistin or nanoemulsion at 2 mg/L for 4 h. The x-axis represents intracellular bacteria after treatments. The y-axis represents the bacterial burden. ANOVA corrected with Tukey’s multiple comparisons test was used to validate the experimental data (****, *p* < 0.0001)

The capacity of the COL-NE to penetrate in macrophages and eradicate intracellular *A. baumannii* was evaluated in in vitro assays. At 3 h post-infection, macrophages were exposed to two treatments, free colistin and COL-NE. The results showed that intracellular bacterial load of *A. baumannii* ATCC19606 strain decreased 3 log_10_ CFU/mL in macrophages treated with COL-NE and 2 log_10_ CFU/mL when the treatment was colistin alone (****, *p* < 0.0001, Fig. 5). Other studies have previously used this methodology to evaluate the uptake of nanocarriers by macrophages and its activity against intracellular pathogens [63, 64]. To the best of our knowledge, no study has described the resistance of *A. baumannii* to eradication when phagocytosed by macrophages in lung infections [65]. An adequate clearance of the phagocytosed bacteria is important to ensure the success of the treatment by totally eradicating the infection.

In clinical practice, an aerosolized administration involves the delivery of the antibiotic to the respiratory tract, thus resulting more effective for the treatment of lung infections [66]. Unfortunately, this hypothetical behaviour has not worked in the case of nebulised colistin. Indeed, free colistin has been found to be toxic upon due to the activation of TGF-β/NOX4 pathway, the production of mtROS and the activation of three major caspases, all this resulting in oxidative stress and mitochondrial apoptotic cell death [13, 14].

**Fig. 6:**
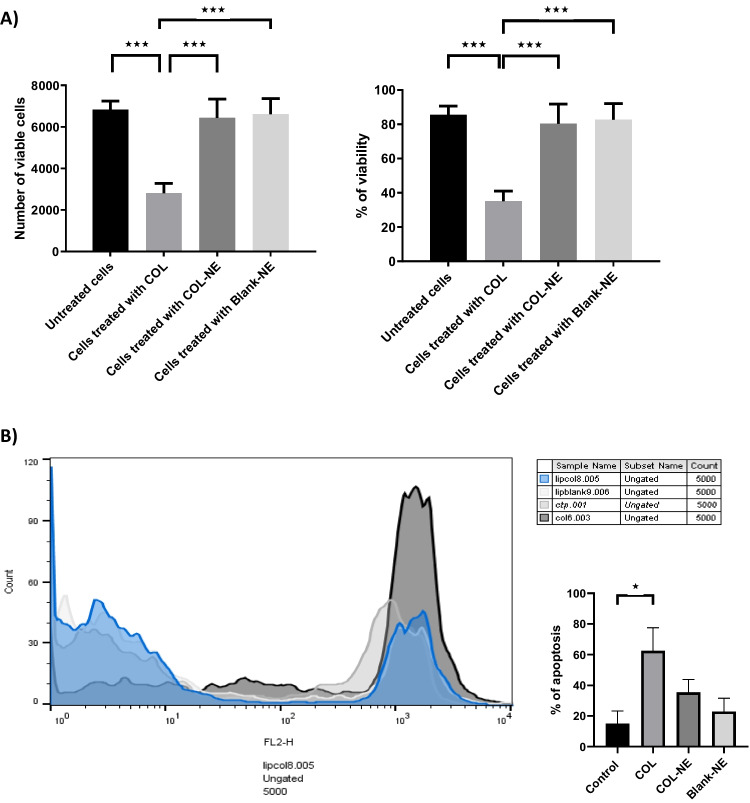
**A**) Cytotoxicity assay in A549 cells performed with CCK-8 kit. ANOVA corrected with Tukey’s multiple comparisons test was used to validate the experimental data (***, *p* < 0.001). **B**) Apoptosis in murine alveolar epithelial cells treated with colistin sulphate or nanoemulsions at 100 mg/L. We observe the cell density stained with IP, i.e., those cells that are in apoptosis. Image extracted from cytometer. B1) Apoptosis in CMT167 cells treated with COL-NE (COL-NE); B2) apoptosis in CMT167 cells treated with blank-NE (blank-NE); B3) apoptosis in untreated CMT167 cells (control); and B4) apoptosis in CMT167 cells treated with colistin (COL). Graph of the obtained results. ANOVA corrected with Tukey’s multiple comparisons test was used to validate the experimental data (*, *p* < 0.05)

In this study, we evaluated the cytotoxicity of COL-NE using both human and murine lung cells. In the colorimetric assay, cells treated with colistin exhibited 35.20 ± 5.8% viability (***, *p* < 0.001), whereas those exposed to COL-NE maintained 80.37 ± 11.3% viability. Treatment with Blank-NE resulted in values comparable to the untreated control (82.75 ± 9.3% vs. 85.45 ± 5%, respectively; Fig. 6A). Flow cytometry confirmed these findings: free colistin induced a significantly higher apoptosis rate (62.55 ± 15.1%, *p < 0.05 compared with COL-NE (35.35 ± 8.6%). In contrast, Blank-NE displayed minimal toxicity, inducing only 22.85 ± 8.7% apoptosis, while the negative control showed 15.07 ± 8.17% (Fig. 6B). These results suggest that our formulations may have reduced cytotoxicity compared to other lipid nanoparticle systems. For instance, a systematic review by Doktorovová et al. reported that lipid nanoparticles typically have an half maximal inhibitory concentration (IC₅₀) range of 0.1–1 mg/mL, in other words, at these concentrations, cell viability is reduced to 50% [67]. In our study, at a nanoemulsion concentration of 0.1 mg/mL (100 mg/L), cell viability reached 80%, corresponding to a 30% enhancement in viability. Furthermore, Pastor et al. evaluated the cytotoxicity of CMS-loaded nanoemulsions in two human lung cell lines, reporting IC₅₀ values of 1.08 mg/mL and 2.59 mg/mL, respectively. In comparison, free CMS exhibited higher toxicity, with IC₅₀ values of 0.006 mg/mL and 0.080 mg/mL in the same cell lines. These findings suggest that CMS-loaded nanoemulsions may offer a safer alternative to free CMS by reducing cytotoxicity while maintaining therapeutic efficacy [68]. Consequently, our results for COL-NE are consistent with previous studies demonstrating the formulation's ability to mitigate drug toxicity.

### Treatment efficacy in a murine pneumonia model

**Fig. 7:**
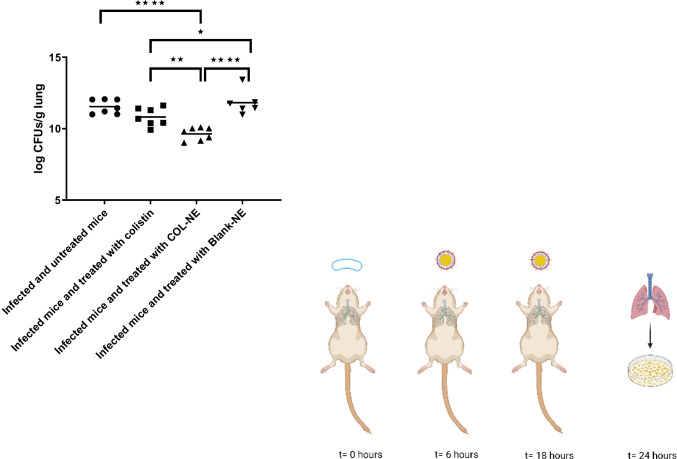
Effect of the therapy with colistin sulphate and nanoemulsions at 2 mg/kg by intratracheal route on the clearance of *A. baumannii* ATCC19606 strain in mouse lung. Each data point represents one animal; the horizontal line in each group depicts the median bacterial burden. ANOVA corrected with Tukey’s multiple comparisons test was used to validate the experimental data (****, *p* < 0.0001)

Based on in vitro data, it was hypothesized that COL-NE could enhance inhalation therapy with free colistin by prolonging lung residence time and maintaining concentrations above the MIC for improved therapeutic efficacy [32]. To test the hypothesis, a murine pneumonia model was used to assess the in vivo antimicrobial effect of COL-NE (Fig. 7). The dose of colistin used was 2 mg/kg. The dose of colistin into COL-NE, with an initial release of 63.85%, was 1.28 mg/kg, which was increased as the release progresses, reaching 2 mg/kg within the first 24 h. Mice treated with free colistin showed a modest reduction in lung bacterial load (0.75 log₁₀ CFU/mL), while those treated with COL-NE exhibited a significantly greater reduction (1.9 log₁₀ CFU/mL, **** *p* < 0.0001) compared to untreated controls. As expected, blank-NE did not exhibit antimicrobial activity. The higher antimicrobial activity observed in the lung for COL-NE as compared to the one reported for Pastor M. et al. was aligned with an improved eradication of the biofilm and targeted access to bacteria. It is worth noting that in the study by Pastor M. *et al*., the evaluation was conducted against *P. aeruginosa*. Intratracheal administration of the CMS nanoemulsion at 2.8 mg/kg (every 12 or 24 h) achieved bacterial loads comparable to free CMS at 15 mg/kg every 12 h, demonstrating greater efficacy at a lower dose. In our study, COL-NE reduced bacterial loads by 1.9 log CFU/g, exceeding the 1.02 log CFU/g reduction reported by Pastor M. et al., highlighting the improved antibacterial performance of our nanoemulsion [69].

In brief, these results report for the first time the efficacy of COL-NE following pulmonary administration and provides insights into their mechanism of action.

## Conclusions

This study reports the design, characterization, and evaluation of a novel colistin-loaded nanoemulsion (COL-NE) with enhanced activity against *A. baumannii*. Inhalation of COL-NE improved therapeutic efficacy in a murine pneumonia model. The formulation’s partial charge neutralization contributed to lower in vitro toxicity without compromising antibacterial activity, allowing for potentially lower and safer dosing. These findings support COL-NE as a promising inhalable platform to enhance the safety and efficacy of last-resort antimicrobials such as colistin against multidrug-resistant pathogens. Future work should aim to assess its pharmacokinetic profile and to further assess long-term safety, immunogenicity, and efficacy against additional MDR organisms.

## Supplementary Information

Below is the link to the electronic supplementary material.

Supplementary file1 (DOCX 189 KB)

## Data Availability

The datasets generated and/or analysed during the current study are available from the corresponding author upon reasonable request.
