# Supplementary material for: Nanoemulsion-based colistin for pulmonary delivery: Enhanced antibacterial efficacy against Acinetobacter baumannii
Source: Drug Deliv Transl Res. 2026 Mar 30;16(7):2474–87. doi: 10.1007/s13346-026-02083-z (PMC13294188; doi:10.1007/s13346-026-02083-z)
Supplement: Supplementary file 1 — Supplementary file1 (DOCX 189 KB) [file 13346_2026_2083_MOESM1_ESM.docx]

**Supplementary Material**

**Figure S1.** Stability study of both COL NE and blank NE in different media. A) Measurement of COL NE’s size for 24 hours. B) Measurement of blank NE’s size for 24 hours. C) Measurement of COL NE’s polydispersity index for 24 hours. D) Measurement of blank NE’s polydispersity index for 24 hours.

1. C)
2. D)

**Figure S2.** Stability study of colistin nanoemulsion during storage at 4 ºC. Size is represented by blue bars and ζ-potential, by red symbol. The x-axis represents the time of storage.
